# Hybrid Bayesian deep learning model for predicting urban heat island intensity in African cities

**DOI:** 10.1038/s41598-025-13492-4

**Published:** 2025-08-25

**Authors:** D. Lynda, G. Logeswari, K. Tamilarasi, S. Rakesh

**Affiliations:** School of Computer Science and Engineering, Vellore Institute of Technology, Chennai, Tamil Nadu 600127 India

**Keywords:** Urban heat islands, Surface urban heat island, Bayesian neural networks, Climate resilience, Urban sustainability, Remote sensing, Machine learning, Climate sciences, Climate-change impacts

## Abstract

Urban Heat Islands (UHIs), in which urban areas have higher temperatures than adjacent rural areas, lead to increased energy demand, poor air quality, and greater public health risks. These impacts are particularly significant in rapidly urbanizing regions of Africa, where climate-adaptive infrastructure has yet to be fully established. Here, we propose a hybrid predictive model derived from Bayesian Neural Networks (BNNs) for deep learning-based forecasting of Surface Urban Heat Island (SUHI) intensities across the African continent. The model employs attention mechanisms and Gradient Boosting Regressors (GBRs), using global climate and urban datasets from over 10,000 cities, incorporating variables such as land surface temperature, land use, and population density. This hybrid ensemble approach outperforms conventional deep learning methods, such as Convolutional Neural Networks (CNN) and Long Short-Term Memory networks (LSTM), when applied to structured climate data. The model was validated using ten-fold cross-validation, yielding a Mean Absolute Error (MAE) of 1.47 °C, Mean Squared Error (MSE) of 3.56 °C^2^, and an R^2^ score of 0.84. Furthermore, it improved the overall accuracy of trained models by 12.3% in suburban areas and achieved an 8.6% reduction in MAE compared to baseline neural architectures. These findings highlight the value of uncertainty-aware and interpretable machine learning tools for climate adaptation. The proposed model can support urban planners in mitigating the thermal burden of rapidly growing urban populations in Africa, ultimately contributing to more livable, climate-resilient cities in the future.

**Supplementary Information:**

The online version contains supplementary material available at 10.1038/s41598-025-13492-4.

## Introduction

The rapid pace of urbanization worldwide has brought numerous socioeconomic benefits but has also triggered significant environmental challenges. Among these, the UHI effect has emerged as a particularly pressing issue for urban planners, policymakers, and environmental scientists^1^. UHI refers to the phenomenon in which urban areas experience significantly higher temperatures than their surrounding rural landscapes^2^. This temperature difference arises primarily because urban environments replace natural vegetation with impervious surfaces such as concrete, asphalt, and buildings, which absorb and retain heat more effectively^3^. Additionally, intensive human activities such as transportation, industrial processes, and energy consumption release substantial amounts of anthropogenic heat^4,5^. These factors collectively contribute to localized warming at the microclimate scale, exacerbating the impacts of global climate change on urban areas.

The consequences of UHIs are extensive and multifaceted. Elevated urban temperatures increase energy demand for cooling, which places additional stress on power grids and raises greenhouse gas emissions. Poor air quality, worsened by higher temperatures, contributes to respiratory and cardiovascular health problems among urban populations, disproportionately affecting vulnerable groups such as the elderly and children^6,7^. Moreover, UHIs alter urban hydrology and exacerbate heat stress, influencing patterns of human behaviour and the dynamics of urban ecosystems. Recognizing these impacts, many cities have made UHI mitigation a priority within broader climate resilience and sustainability frameworks^6^.

In response to growing awareness, research efforts have focused on understanding the mechanisms behind UHI formation and exploring a range of mitigation strategies. Techniques such as installing green roofs, using cool and reflective materials for pavements and rooftops, enhancing urban forestry, and restoring water bodies have shown promise in lowering urban temperatures^8^. These interventions work by increasing surface albedo (reflectivity), enhancing evapotranspiration, and providing shading, which together reduce heat accumulation. To support targeted and effective urban heat management, advanced spatial analysis tools such as Urban Climatic Maps (UCMaps) and Geographic Information Systems (GIS) have been developed and widely adopted. These tools enable detailed mapping of thermal and wind patterns at fine spatial scales, allowing planners to identify heat hotspots and prioritize cooling measures. Notably, more than 15 countries have integrated such spatial tools into their real-time urban planning policies, signaling growing institutional recognition of the importance of UHI mitigation.

Despite these advances, accurately predicting how UHIs will evolve with ongoing urban growth and how different mitigation strategies will perform remains a significant scientific challenge^9^. UHIs result from complex interactions among numerous factors, including land use and land cover (LULC) changes, urban morphology (such as building density, height, and layout), canopy cover, anthropogenic heat emissions, and meteorological conditions such as wind patterns and humidity. Traditional numerical weather prediction models, such as the Weather Research and Forecasting (WRF) system, have been adapted for urban climate studies and have provided valuable insights through scenario experiments^10^. However, the multi-scale nature of UHIs, which vary across space and time, and their nonlinear interactions often exceed the representational capacity of conventional models. Moreover, these models typically produce deterministic outputs without explicitly addressing the uncertainties inherent in urban environmental data, which limits their usefulness for risk-informed urban planning.

There is a pressing need for modelling approaches that can simultaneously address the complexity, heterogeneity, and uncertainty of urban climate systems. Specifically, models must be capable of integrating diverse data sources, ranging from satellite remote sensing and ground-based sensors to socio-economic datasets, and capturing the nonlinear relationships and feedback mechanisms between variables^11,12^. Additionally, providing uncertainty quantification in predictions is essential for planners to assess confidence levels and make informed decisions.

To address these challenges, this research proposes a novel hybrid modelling framework that combines BNNs enhanced with attention mechanisms and a GBR. BNNs are a probabilistic extension of CNN, providing the capability to model uncertainty in both data and model parameters. This enables BNNs to generate prediction intervals rather than single point estimates, which is essential when working with noisy, incomplete, or heterogeneous urban datasets. The attention mechanism incorporated into the BNN architecture allows the model to automatically identify and focus on the most relevant spatial and temporal features, thereby improving interpretability and prediction accuracy. Complementing the BNN, the GBR is used to model residual errors and correct biases, leveraging its strength in capturing complex, nonlinear patterns and iteratively reducing errors.

Our methodology integrates in situ air temperature measurements with a comprehensive set of predictor variables, including land cover types, population density, urban geometric metrics, traffic intensity and climate variables. Bayesian inference techniques are employed to model the spatiotemporal evolution of UHI intensity, explicitly accounting for uncertainty in parameter estimates and predictions. While the primary focus of this study is on African urban centers, where data scarcity and rapid urbanization amplify UHI risks, the underlying modelling framework has been initially tested and validated using data from the Jing-Jin-Ji metropolitan region in China, spanning a 15-year period (2003–2018). This initial validation demonstrated the model’s ability to capture both intra-urban temperature variability and the influence of urban expansion, land use change and vegetation dynamics. The integration of satellite-derived products such as Moderate Resolution Imaging Spectroradiometer (MODIS) land surface temperature and Landsat-based imperviousness indices further enriches the spatial characterization of UHI effects, allowing detailed assessment of urban cores and peripheral suburban zones.

The hybrid BNN-GBR ensemble approach offers multiple advantages. The probabilistic outputs of the BNN enable planners to understand and quantify the uncertainty in temperature projections, while the GBR component enhances prediction robustness by capturing residual nonlinearities. Initial comparisons indicated that the GBR outperformed other ensemble methods, such as Random Forests (RF), in residual modelling for this dataset. Together, the models produce fine-scale spatial maps of UHI intensity and its seasonal variations, which are vital for urban heat risk assessment and management. Table 1 lists the abbreviations and acronyms used in this research.

**Table 1:** Abbreviations used in this research work.

Abbreviation	Definition
BNN	Bayesian neural network
CNN	Convolutional neural network
XGBoost	Extreme gradient boosting
GBR	Gradient boosting regressor
GIS	Geographic information system
LCZ	Local climate zone
LST	Land surface temperature
LSTM	Long short-term memory
LULC	Land use and land cover
MAE	Mean absolute error
MODIS	Moderate resolution imaging spectroradiometer
MSE	Mean squared error
NDBI	Normalized difference built-up index
NDVI	Normalized difference vegetation index
RF	Random forest
RMSE	Root mean squared error
SUHI	Surface urban heat island
TSUHI	Tropical surface urban heat island
UCMap	Urban climatic map
UHI	Urban heat island
ULST	Urban expansion to land surface temperature
VIF	Variance inflation factor
WRF	Weather research and forecasting

### Novelty

This research introduces a series of key innovations that distinctly set it apart from existing studies on UHI prediction and modeling:

- Introduces a novel ensemble model combining BNNs with attention mechanisms and GBRs for accurate and interpretable SUHI forecasting.
- Employs probabilistic modeling to quantify uncertainty in predictions, supporting risk-informed climate adaptation strategies.
- Uses GBR to model and correct residual errors from the BNN, enhancing robustness and capturing complex nonlinear patterns.
- Applies the model across more than 10,000 African cities using global climate and urban datasets, making it one of the largest-scale UHI prediction studies to date.
- Achieves improved accuracy over conventional deep learning models, with a 12.3% performance gain in suburban areas and an 8.6% reduction in MAE.
- Validated through ten-fold cross-validation, ensuring reliability and adaptability across diverse urban morphologies.
- Generates high-resolution, seasonally resolved SUHI risk maps to support targeted, climate-resilient urban planning interventions.

The remainder of the paper is organized as follows: Section II reviews existing literature on UHI mechanisms, modeling approaches, and mitigation strategies. Section III describes the proposed system architecture, data sources, feature engineering, and modeling procedures. Section IV presents experimental results, model evaluation, and interpretation of key findings. Section V concludes the study and summarizes the main contributions. Section VI highlights potential directions for future research and model enhancement.

## Literature survey

The study of urban climate is crucial for understanding the trends and impacts of climate change in cities, particularly in relation to the UHI phenomenon. This section provides an extensive review of existing research on UHIs, including policy frameworks for UHI mitigation, data analytical approaches, and simulation and machine learning tools for exploring and predicting UHI effects.

### UHI patterns across African regions

*North Africa*: The UHI phenomenon, a critical consequence of rapid urbanization and land-use transformation, has attracted increasing scholarly attention across the African continent due to its profound implications for urban sustainability, public health, and climate resilience. In North African regions characterized by arid and semi-arid climates, Abulibdeh analyzed the pronounced UHI effects in cities such as Cairo and Algiers^1^. The study attributed the high nocturnal UHI intensity to minimal vegetation cover, highly absorptive building materials, and dense urban forms that trap solar radiation. In these climates, the lack of evapotranspiration and reflective surfaces intensifies heat accumulation during the day and limits nighttime cooling, resulting in elevated surface and ambient temperatures in urban centers compared to surrounding rural areas.

*West Africa*: In West Africa, research conducted in Lagos employed multi-temporal satellite remote sensing data (Landsat and MODIS) to assess the spatial distribution of Land Surface Temperature (LST) and its relationship with land cover dynamics^3^. The findings demonstrated a strong positive correlation between impervious surface expansion and increases in LST. Urban growth, driven by population pressures and infrastructural development, has led to the conversion of vegetated and wetland areas into asphalt and concrete surfaces, which have low albedo and high heat storage capacity. Notably, areas with higher built-up density, such as central Lagos, recorded LST values exceeding 40 °C during dry seasons, revealing significant intra-urban thermal disparities. Guo et al.^13^ also estimated the relative contributions of global climate change and urban expansion to land surface temperature (ULST) increases in Lagos using MODIS imagery and regression decomposition. Their estimates showed that urbanization accounted for 61% of ULST increases, particularly during dry daytime periods, confirming the significant role of local land-use policies in reducing UHI.

*East Africa*: In East Africa, a study in Cairo investigated the influence of UHIs on urban energy demand using thermal imaging and energy consumption data^8^. Buildings located in high-UHI zones were found to consume more electricity for cooling, especially during the summer months, thereby reinforcing the feedback loop between UHIs and energy stress. In contrast, studies in Nairobi demonstrated that urban green spaces such as parks and tree-lined streets provide significant localized cooling benefits^9^. Using thermal infrared imagery and GIS-based spatial analysis, researchers observed a temperature difference of up to 5 °C between green and built-up zones, highlighting the crucial role of vegetation in moderating microclimates and enhancing thermal comfort. These findings underscore the importance of strategically planned green infrastructure. The proposed model offers a probabilistic basis for identifying priority sites for interventions such as targeted green infrastructure or zoning reforms, aiming to maximize cooling and resilience benefits in high-risk zones. This approach is especially relevant for fast-growing suburban regions, where unchecked expansion contributes to further heat emissions and exacerbates the urban thermal burden.

A longitudinal study in Addis Ababa combined Landsat TM/ETM + data with in situ temperature readings to explore the evolution of UHIs^14^. Informal settlements were found to experience more severe heat stress due to poor ventilation, limited tree cover, and unpaved surfaces. The study advocated for the integration of green belts and artificial lakes as measures to counteract rising urban temperatures. Meanwhile, simulations in Harare using cellular automata and Markov Chain models projected significant UHI intensification under various urban growth scenarios, particularly in the absence of greening policies^15^. Garuma^16^ assessed Tropical Surface Urban Heat Island (TSUHI) profiles in capital cities across East Africa from 2000 to 2020 using MODIS and climatological data. He reported that TSUHI intensities correlated with vegetation cover, population density, and drought vulnerability, ranging from 1 °C in Dodoma to over 8 °C in Khartoum.

*Central and Southern Africa*: In Johannesburg, a study using Landsat and GIS tools identified persistent thermal hotspots in the central business district and industrial zones, attributed to metal roofs and asphalt surfaces^17^. Additional research explored the effect of urban geometry on pedestrian thermal comfort. Simulations showed that narrow streets with low sky view factors trap heat, but vertical greenery systems and shaded arcades can significantly reduce thermal stress^12^. The relationship between land cover and surface temperature was confirmed in Addis Ababa, where forested and agricultural areas consistently recorded lower temperatures than built-up zones^18^. The introduction of Local Climate Zones (LCZs) allowed standardized classification of urban environments for more accurate UHI assessments across different African cities^19^. In Kampala, localized climatic changes linked to UHI were observed, including increased frequency of heatwaves in densely populated slums^20^.

*Island Cities*: On Réunion Island, Lefevre et al.^21^ adapted a LCZ framework for urban heat modelling across the island. UHI intensity reached a maximum of 4.14 °C in dense urban microclimates. This study is an important example demonstrating the role of spatial zoning in urban planning and the importance of climate-sensitive gradients of heat in tropical island urban environments.

### Predicting urban heat islands across continents and existing models

Recent research has focused on developing predictive models to understand and mitigate UHI effects, employing various methodologies from numerical simulations to machine learning approaches.

#### Continental variations in UHI research

*Asian Cities*: Asian cities present unique UHI characteristics due to their dense populations and rapid urbanization. Studies across 100 Asian and Australian cities found UHI magnitudes varying between 0.4 K to 11.0 K, with particularly intense effects in tropical and subtropical cities where UHI superimposes on already hot climates^22^. Unlike temperate cities where maximum UHI intensity typically occurs at night, many Asian cities like Tokyo and Beijing show peak intensity during daytime due to high anthropogenic heat fluxes (exceeding 400 W/m^2^ in Tokyo). Research in Southeast Asian cities like Bangkok demonstrates UHI’s significant impact on household energy consumption, with cooling degree days strongly correlated with increased electricity usage^23^. Machine learning approaches in Mansoura, Egypt predicted that without intervention, over 40% of the city could experience land surface temperatures above 45 °C by 2031 due to urban expansion^24^.

*European Cities*: European research has emphasized the integration of UHI modeling with urban planning. The TERRA_URB scheme, tested in cities like Turin, Naples, and Moscow, successfully reproduced UHI effects in the COSMO meteorological model by accounting for urban geometry and heat fluxes^25^. Studies in Mediterranean cities revealed an intensification of surface UHI over 18 years, with temperature increases ranging from + 0.412 K in Marseille to + 0.92 K in Cairo^26^. Vienna-based research highlighted how passive design measures in buildings could mitigate UHI by reducing air conditioning waste heat^26^. The urban canyon effect is particularly pronounced in Europe’s historic cities with narrow streets and tall buildings.

*American Cities*: North American studies have contributed significantly to UHI measurement methodologies, including the UHI Index developed by California EPA, which sums temperature differences in degree-Celsius-hours. Research in Phoenix identified pavement infrastructure as a primary contributor to afternoon urban heat^27^. Machine learning models analyzing 216 global cities found that while wind speed significantly mitigates UHI intensity, temperate climate cities show more pronounced effects than tropical ones 8. Coastal proximity was identified as another important factor in reducing UHI effects in American cities^28^.

### Evolution OF UHI prediction models

*Early numerical models*: The foundation for UHI modeling was established by Leonard O. Myrup in 1969 with a comprehensive numerical treatment using energy budget approaches. These early models identified reduced evaporation and thermal properties of urban materials as dominant parameters but required extensive city characteristic data^29^. The limitations of these static models became apparent as urban environments grew more complex.

*Modern simulation environments*: Contemporary models like ENVI-met simulate all interactions between buildings, ground surfaces, plants, and ambient air. The WRF model has been widely adapted for urban climate studies, while Computational Fluid Dynamics approaches address microclimate variations^30^. The SLEUTH model has been employed to investigate UHI effects on global environmental change. These models have improved significantly but still face challenges in computational efficiency and data requirements.

*Machine learning advancements*: Recent years have seen a surge in machine learning applications for UHI prediction. A global study of 216 cities using Support Vector Regression achieved a mean absolute error of 0.86 °C by integrating climate, economic, population, and land use data^28^. Artificial Neural Networks and Cellular Automata have been successfully applied to predict land use/cover changes and their thermal impacts, as demonstrated in Mansoura City^24^. These approaches excel at handling complex, sparse datasets and identifying intricate patterns that traditional models might miss.

### Public health and socioeconomic impacts

Extending further into public health, a study in Accra explored the link between UHI-induced temperature extremes and heat-related mortality^6^. The research showed that elderly populations and individuals with pre-existing cardiovascular conditions were particularly vulnerable during heatwaves exacerbated by UHI effects. The lack of green infrastructure and inadequate housing insulation in informal settlements contributed to increased indoor temperatures, highlighting the need for urgent policy interventions focused on climate-sensitive housing design and heat-health warning systems.

Gyile et al.^31^ used a time-series Landsat analysis across Greater Accra to demonstrate a 4 °C increase in LST in urban areas from 1991 to 2020, while Normalized Difference Vegetation Index (NDVI) showed a steep decline. The study emphasized the strong inverse correlation between vegetation cover and surface temperature and highlighted NDVI’s importance as a critical indicator of urban cooling within urban morphology. The authors suggested the need to integrate vegetated buffers along streets, plant shade trees, and enforce land-use zoning to reduce the impacts of thermal stress in urban neighborhoods in the future.

### Air quality, climate interaction, and theoretical foundations

In Dar es Salaam, the UHI phenomenon was investigated in relation to atmospheric pollution and urban geometry^10^. A positive relationship was found between increased ambient temperature and the concentrations of ground-level ozone and fine particulate matter. Compact urban forms with poor air circulation exacerbated both heat accumulation and pollutant stagnation. A foundational contribution to UHI theory came from Oke, who identified critical variables such as surface roughness, sky view factor, and anthropogenic heat release^11^. This framework remains central to UHI research globally. Building on this, researchers have demonstrated the utility of high-resolution thermal imagery and remote sensing indices such as NDVI, Normalized Difference Built-up Index (NDBI), and LST to quantify spatial UHI variability in African cities^32^.

The interaction between UHI and air quality has been examined in other contexts as well, showing that UHI influences boundary layer dynamics and pollutant dispersion^33^. Although outside the African context, studies of UHIs in shrinking cities have highlighted that even depopulated urban areas can retain heat due to existing built infrastructure^34^. Research into climate change awareness revealed limited understanding of UHI among university students in Nigeria, indicating a gap in environmental education^35^. Adding a further layer of complexity, some studies have shown that UHI can induce localized convective activity, potentially affecting rainfall patterns^36^. Comparisons between compact and sprawling cities have found that low-density urban expansion with minimal vegetation cover leads to higher UHI intensity and increased vulnerability ^7^.

### Mitigation strategies and urban design

Reviews of UHI mitigation strategies conducted over the past three decades have categorized interventions such as high-reflectance materials and green infrastructure by their effectiveness and cost, highlighting cool roofs and vegetative solutions as particularly impactful^37^. A broader synthesis of UHI causative factors and mitigation methods has emphasized the importance of systemic planning and multi-sector collaboration^26^. Studies focusing on roofing materials have demonstrated that green roofs and reflective coatings significantly reduce building heat gain^38^. Other research in high-density Asian cities has corroborated these findings, noting that urban form and material choice are pivotal in shaping thermal profiles^39,40^. Rooftop gardens have also been found to provide measurable cooling and insulation benefits in tropical settings^41^, while vertical greening systems have proven particularly effective in narrow, high-rise urban corridors^42^.

Collectively, these studies offer a comprehensive understanding of UHI dynamics in African urban contexts, revealing the intertwined influences of land use, climate, urban morphology, and socio-economic structures. The evidence supports the need for integrated, multi-disciplinary approaches to mitigate UHI effects through sustainable design, green infrastructure, and informed policies tailored to the unique environmental and demographic conditions of African cities.

## Proposed architecture

**Fig. 1:**
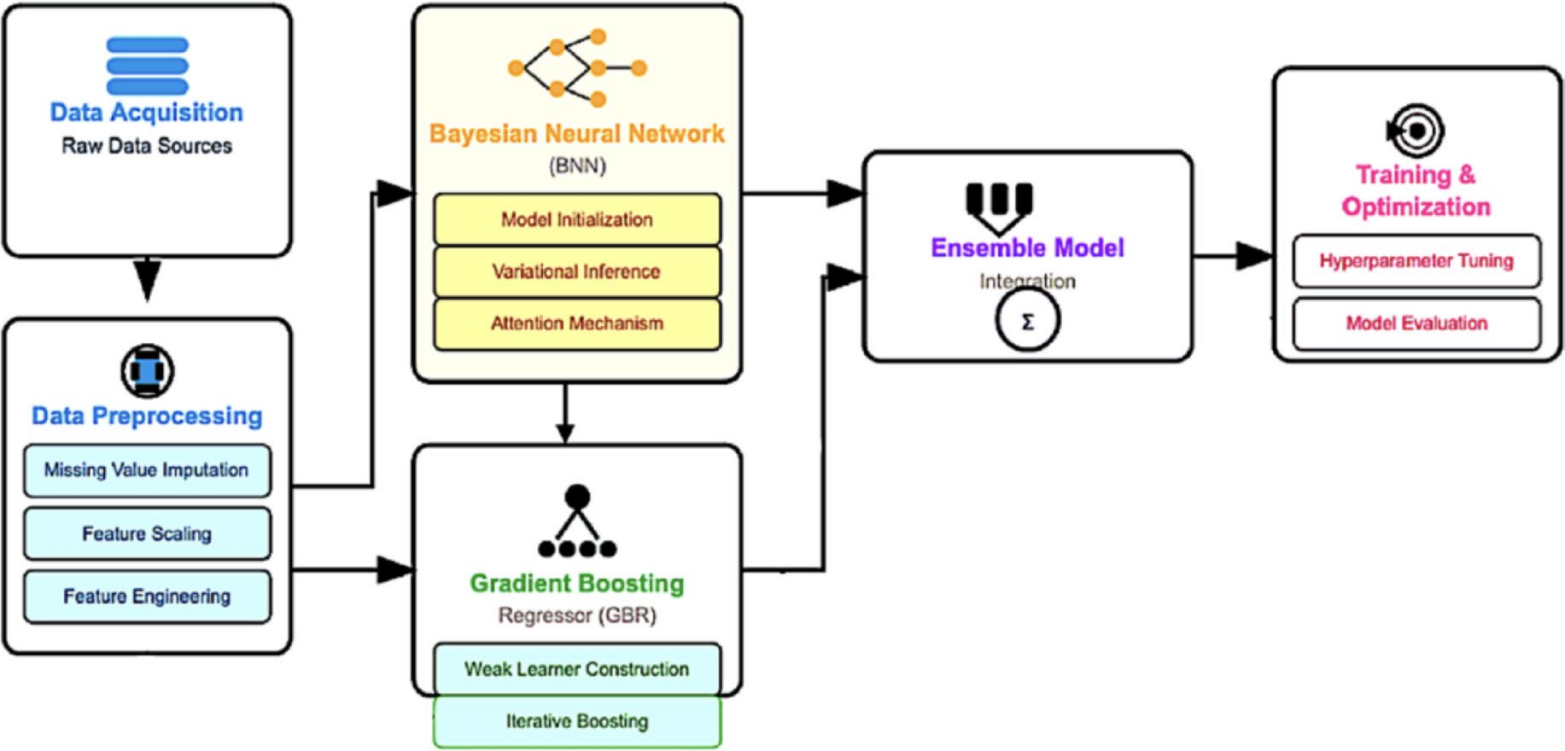
A diagrammatic overview of the proposed system.

This research aims to employ advanced machine learning models to predict the UHI effect. The methodology is carefully designed to address the inherent challenges of UHI data, which span multiple urban centers across both spatial and temporal dimensions. The proposed framework adopts a powerful yet interpretable predictive modeling approach by integrating BNNs, attention mechanisms, and GBRs. The novelty of this integration lies in its synergistic capabilities: BNNs offer reliable uncertainty quantification, which is critical for climate-related forecasting; the attention mechanism improves interpretability by identifying and emphasizing the most influential spatial and temporal features; and GBRs effectively capture complex non-linear interactions, enhancing prediction accuracy by modeling residual errors. This hybrid approach is preferred over conventional deep learning architectures such as s or LSTM networks, particularly for tabular climate and urban morphology datasets. Unlike end-to-end deep learning models that often require extensive spatio-temporal structuring, this ensemble method provides a balanced solution by combining strong predictive performance with transparent insights into the underlying factors driving UHI patterns. The architecture of the proposed system is illustrated in Fig. 1.

### Data source

The dataset used in this research is sourced from the Global UHI Dataset, made publicly available by the Center for International Earth Science Information Network (CIESIN). It integrates data from remote sensing instruments (such as MODIS) and other geographic and socioeconomic sources to quantify UHI intensity across urban and rural regions globally. The data includes essential parameters such as daytime and nighttime land surface temperatures (LST), spatial coordinates (latitude, longitude, altitude), population distribution, and land use characteristics.

*Database*: The dataset contains georeferenced variables that are needed to model the UHI effect across the globe.

#### Dependent variable

- D_T_DIFF: Daytime Urban Heat Island intensity, is defined as the temperature difference between an urban area and the surrounding rural area during the daytime, which is the variable that was predicted in this study.
- N_T_DIFF: Nighttime Urban Heat Island intensity, is defined as the temperature difference between urban and rural areas during nighttime. This variable is included in this study as exploratory analysis but is not a variable that is predicted.

#### Independent variables

- ISOURBID: A unique identifier character created from joining the ISO3 country code to the urban region id number.
- ISO3: Three letter country code from the ISO standard.
- URBID: Number of urban region ID within country.
- NAME / SCHNM: Formatted official city or area names.
- ES90POP, ES95POP, ES00POP: Estimate population values for the years 1990, 1995, & 2000.
- SQKM_FINAL: Area of the urban extent in sq. km.
- URB_D_MEAN: Average maximum land surface temperature during the daytime in urban area.
- BUF_D_MEAN: Average maximum land surface temperature during the daytime in the surrounding 10 km’s buffer.
- URB_N_MEAN: Average minimum land surface temperature during the night in urban area.
- BUF_N_MEAN: Average minimum land surface temperature during the night in surrounding 10 km’s buffer.
- LATITUDE/LONGITUDE: Geographical coordinates indicating the centroid of the urban region.

#### Derived variables

- Density: Population divided by its respective urban area devises the population estimate by the corresponding urban area to account for population occurrences in a unit area.
- Temperature Difference Over Time: This variable encapsulates the differences in surface temperature between the most recent decade and past decades, and is created to represent warming trends associated with urbanization.

These variables provide a complete representation of urban morphology, thermal environment, and demographic pressures affecting UHI intensity. This database will facilitate modeling of the UHI phenomenon spatially and temporally.

#### Urban and rural area classification

The key dependent variable in this task is UHI intensity (ΔT), which measures the thermal difference between urban and nearby rural regions. To calculate UHI intensity (ΔT), urban and rural regions were distinguished based on vegetation cover using impervious surface fractions from Landsat-based remote sensing products in the Global UHI Dataset. In this project, a region with impervious surface fraction ≥ 50% was considered as urban and a region with an impervious surface fraction ≤ 10% was considered as rural. These thresholds represent common thresholds in UHI research and provide an approach that is consistent and scalable across the globe. We chose to use the impervious surface thresholds as they are publicly available in the dataset and they have more spatial coverage than Local Climate Zone (LCZ) classification. UHI intensity (ΔT) is formally defined by Eq. (1):

1 $${\Delta T} = T_{urban} - T_{rural}$$

where ${T}_{urban}$ denotes the land surface temperature in urban zones, and ${T}_{rural}$ denotes the corresponding temperature in nearby rural areas. This variable captures the localized temperature anomaly attributed to urbanization.

### Data preprocessing

*Missing value imputation*: Preprocessing begins by handling missing or incomplete data to ensure the integrity of the dataset. For continuous variables such as land surface temperature and vegetation indices, missing values are filled using mean imputation, where each missing entry is replaced with the average value of the observed data. This helps maintain the overall distribution of the data without introducing bias. For temporal variables, such as temperature trends observed over time, linear or spline interpolation methods are applied. These approaches estimate the missing values based on surrounding time points, thereby preserving the continuity and natural flow of temporal patterns essential for accurate trend analysis.

*Feature scaling*: To improve the efficiency and stability of model training, especially in algorithms that rely on gradient-based optimization, feature scaling is applied. Min–Max normalization is used to rescale the features so that all values fall within a uniform range, typically between zero and one. This ensures that no single feature dominates the learning process due to a larger numerical range, which could distort model predictions. Uniformly scaled features allow the model to learn patterns more effectively and lead to faster convergence during training.

*Feature engineering*: To enrich the dataset with more informative attributes, additional derived features are introduced. One such feature is population density, which is calculated by dividing the total number of people by the corresponding land area of a spatial region. This provides insight into human habitation intensity and its potential impact on environmental or climatic patterns. Another important feature is the temperature gradient over time, which represents the change in land surface temperature between two different time periods. This captures long-term warming trends or cooling shifts, offering valuable context for analyzing phenomena such as climate change or urban expansion.

### BNN with attention mechanism

BNN is a probabilistic model where the weights of the neural network are treated as random variables with prior distributions. This allows the model not only to predict an output but also to estimate the uncertainty in its prediction. The general form of the BNN output is given in Eq. (2):

2 $$y = f\left( {X;W} \right) + \epsilon$$

where y is the predicted UHI intensity, X is the input feature vector, W∼ N (0, I) is the weight vector assumed to be drawn from a standard multivariate normal distribution, and ϵ∼ N (0, σ^2^) represents Gaussian noise.

Since computing the exact posterior distribution of weights given data D, denoted P(W∣D), is computationally intractable, variational inference is used to approximate it. This approach replaces the true posterior with a simpler distribution q(W), and minimizes the Kullback–Leibler divergence between them.

3 $$\min_{q} {\text{KL}}\left( {{\text{q}}\left( {\text{W}} \right)} \right){\text{||P }}\left( {{\text{W }}|{\text{ D}}} \right)$$

Equation (3) transforms Bayesian inference into an optimization problem, allowing scalable and tractable training of deep probabilistic models.

To enhance interpretability and focus on the most relevant features, an attention mechanism is integrated. It assigns a learnable weight to each input feature. The attention score for feature i, denoted as ${e}_{i}$ is calculated using a feedforward layer followed by a non-linearity as shown in Eq. (4).

4 $$e_{i} = \tanh \left( {W_{e } x_{i} + b_{e} } \right)$$

Then, the attention weight ${\alpha }_{i}$ is computed as shown in Eq. (5) via a softmax function across all feature scores:

5 $$\alpha_{i} = \frac{{\exp \left( {e_{i} } \right)}}{{\mathop \sum \nolimits_{j} \exp \left( {e_{j} } \right)}}$$

Here, ${x}_{i}$ is the i^th^ feature value, ${W}_{e}$ and ${b}_{e}$ are trainable parameters, and ${\alpha }_{i}$ reflects the relative importance of the feature in predicting y.

### Gradient boosting regressor

GBR is used as a complementary model to capture residual errors not modeled by BNN. It is a tree-based ensemble method that builds a sequence of decision trees, each trained to correct the errors of the previous ensemble. The prediction at the *m*th iteration is defined by Eq. (6):

6 $$F_{m} \left( x \right) = F_{m - 1} \left( x \right) + \alpha . h_{m} \left( x \right)$$

where ${F}_{m}\left(x\right)$ is the ensemble prediction at stage m, ${h}_{m}\left(x\right)$ is the new weak learner (a decision tree), and α is the learning rate that controls the contribution of ${h}_{m}$. The model iteratively minimizes the gradient of the loss function with respect to the prediction.

### Ensemble model output

To combine the strengths of BNN’s probabilistic predictions and GBR’s non-linear tree-based learning, the outputs are merged through a weighted ensemble. The final UHI intensity prediction $\widehat{y}$ is computed as shown in Eq. (7).

7 $$\hat{y} = w_{1} \hat{y}_{BNN} + { }w_{2} \hat{y}_{GBR}$$

where $\widehat{y}$ is the final predicted UHI intensity, ${\widehat{y}}_{BNN}$ and ${\widehat{y}}_{GBR}$ are predictions from BNN and GBR respectively, ${w}_{1}$+ ${w}_{2}=1$ are ensemble weights optimized using validation data to maximize generalization.

### Model training strategy

Loss Function for BNN: To train the BNN, regularized mean squared error loss is used as shown in Eq. (8).

8 $$L\left( \theta \right) = \frac{1}{N}\mathop \sum \limits_{i = 1}^{N} \left( {y_{i} - f\left( {x_{i} ;\theta } \right)} \right)^{2} + \lambda \left\| \theta \right\|^{2}$$

where ${y}_{i}$ is the true UHI value for the i^th^ instance, $f({x}_{i};\theta )$ is the model prediction, θ denotes the model parameters (weights), N is the number of training samples, and λ is the L2 regularization coefficient that prevents overfitting by penalizing large weights. This loss ensures that the model not only fits the data but also maintains generalization by discouraging overly large weights.

### Model evaluation

To evaluate the model’s performance and ensure it generalizes well to unseen data, tenfold cross-validation is employed. The dataset is divided into 10 equal parts; the model is trained on 9 parts and validated on the 1 remaining part in each iteration. The average cross-validation error is computed as shown in Eq. (9):

9 $$CV\;Error = \frac{1}{k}\mathop \sum \limits_{i = 1}^{k} L_{i}$$

where k = 10 is the number of folds, and ${L}_{i}$ is the loss on the i^th^ fold. This ensures a robust assessment of the model and reduces variance in performance estimates.


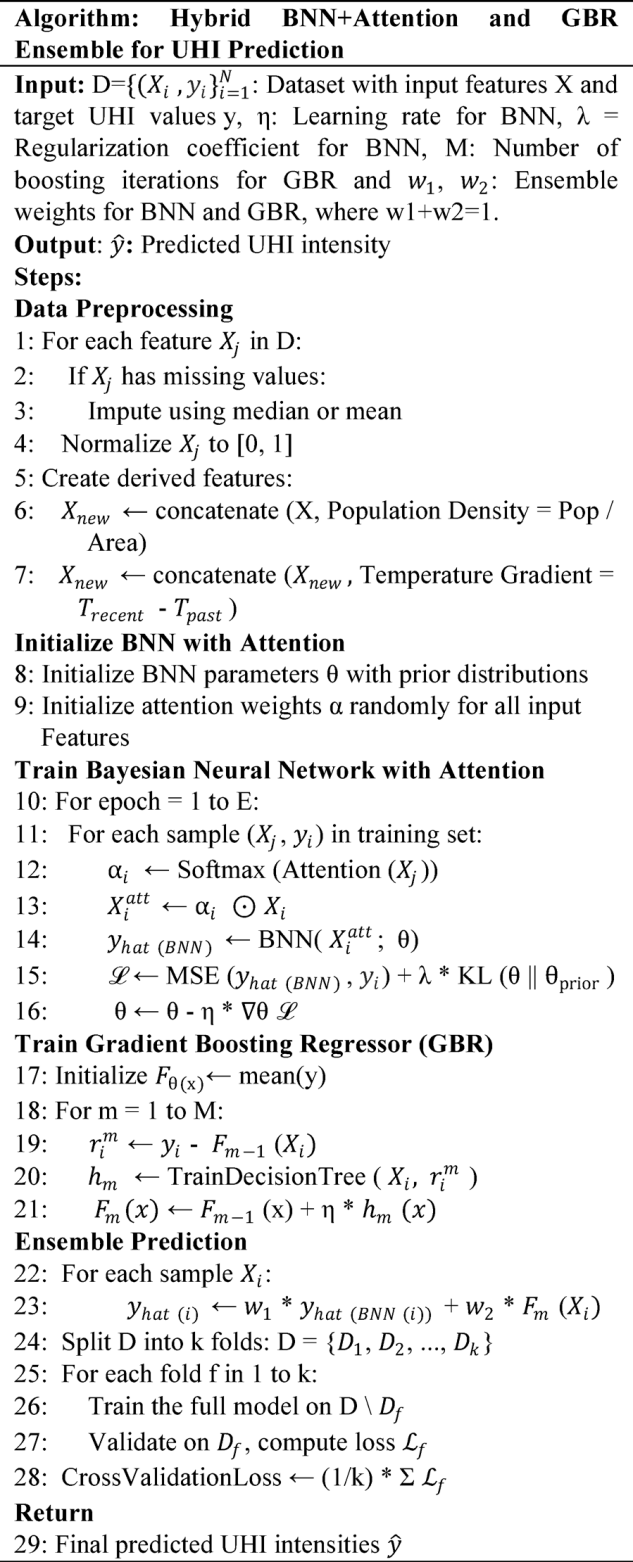


## Results and discussions

### Performance metrics

To evaluate the predictive performance of the models, the following metrics were utilized:

MAE measures the average magnitude of errors in predictions, without considering their direction as shown in Eq. (10).

10 $$MAE=\frac{1}{n} \sum_{L=1}^{n}\left|{y}_{i}-{\widehat{y}}_{\dot{i}}\right|$$

where ${y}_{i}$ and ${\widehat{y}}_{\dot{i}}$ are the observed and expectant values respectively.

MSE penalizes larger errors more heavily due to the square term, making it sensitive to outliers as shown in Eq. (11).

11 $$MSE=\frac{1}{n}\sum_{L=1}^{n}{{(y}_{i}-{\widehat{y}}_{\dot{i}})}^{2}$$

Coefficient of Determination (${R}^{2}$):

12 $${R}^{2}=1- \frac{\sum_{L=1}^{n}{{(y}_{i}-{\widehat{y}}_{\dot{i}})}^{2}}{\sum_{L=1}^{n}{{(y}_{i}-\overline{y })}^{2}}$$

where $\overline{y }$ is the average of the observed values. The greater the *R*^*2*^ the better the model which accounts for most of the variations present in the data. Coefficient of Determination is computed as shown in Eq. (12).

### Descriptive statistics

Descriptive analysis was conducted on the selected features and the target variable, D_T_DIFF (daytime temperature difference between urban and rural regions). Measures such as mean, median, minimum, maximum, and standard deviation were used to identify data trends and detect outliers. Data points exceeding three standard deviations from the mean were reviewed, and approximately 0.5% of values were winsorized at the 99.5th percentile to manage extreme values. Additionally, skewness and kurtosis were assessed to understand the distribution shapes and inform the selection of appropriate data transformations. For example, population density variables exhibited high positive skewness (skewness > 1), prompting the application of logarithmic transformation prior to modeling. Table 1 presents the key statistical measures. The mean D_T_DIFF was 3.2 °C with a standard deviation of 1.5 °C, reflecting notable variability. The urban area size (SQKM_FINAL) ranged from 10 km^2^ to over 5000 km^2^, indicating high dispersion across urban regions.

### Correlation matrix

**Fig. 2:**
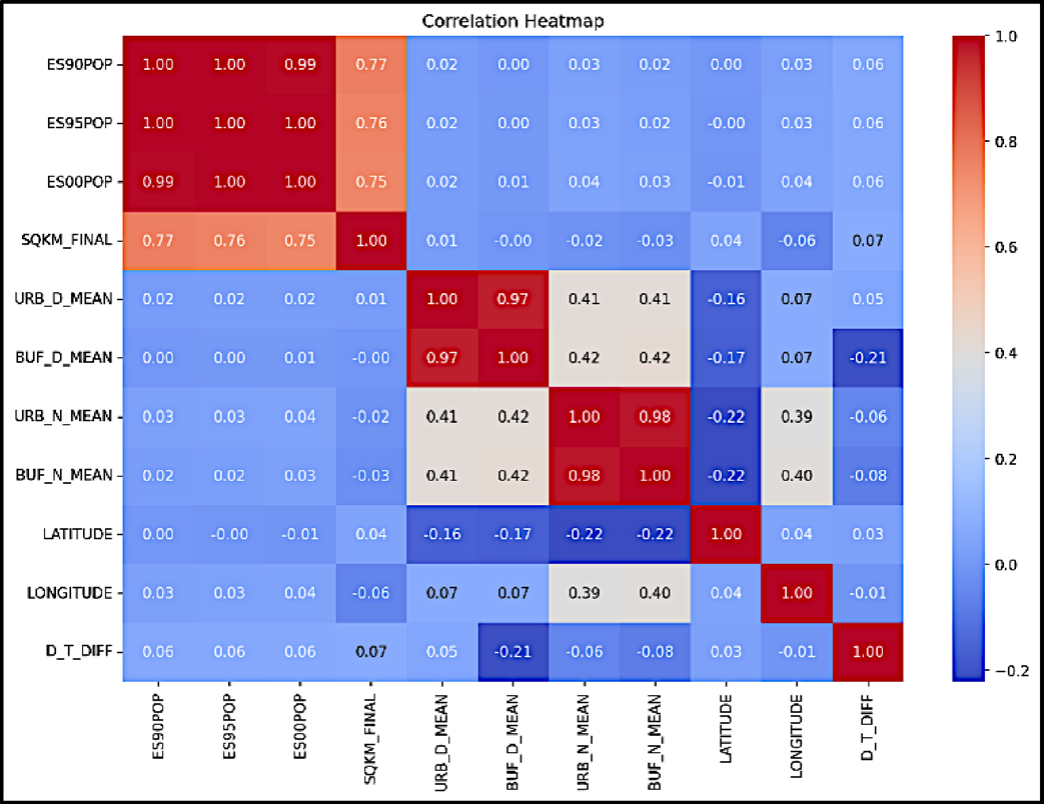
Correlation heatmap of the features.

The correlation matrix is a fundamental tool in feature engineering and model building, used to understand how specific features relate to both the target variable and to each other. A correlation matrix was constructed for all selected features along with the target variable. This matrix includes Pearson correlation coefficients, which indicate the degree of linear association between pairs of variables. For example, a positive correlation was observed between urban area size and temperature difference, suggesting that larger cities tend to exhibit a more pronounced UHI effect. Beyond individual correlations, the matrix also aids in detecting multicollinearity, a condition where two or more features are highly correlated. A threshold of Pearson’s r > 0.8 was used to flag potential multicollinearity. To supplement this, Variance Inflation Factor (VIF) scores were calculated for each predictor, with features having VIF > 5 marked for review. For instance, a high correlation (r = 0.92, VIF = 6.5) was found between population in 1990 and population in 1995. Although population in 1995 showed a slightly higher correlation with the target variable, population in 1990 was retained, as the model demonstrated robustness to multicollinearity. Table 2 presents the numerical values of the correlation matrix, while a heatmap visualization is shown in Fig. 2 to provide a clearer overview of the relationships.

**Table 2:** Descriptive statistics of the features.

	Population in 1990 (ES90POP)	Population in 1995 (ES95POP)	Population in 2000 (ES00POP)	Urban area (sq. km)	Urban daytime mean temp (°C)	Buffer daytime mean temp (°C)	Urban nighttime mean temp (°C)	Buffer nighttime mean temp (°C)	Latitude (decimal degrees)	Longitude (decimal degrees)	Daytime temp difference (°C)
Count	31500.00	31500.00	31500.00	31500.00	31492.00	31500.00	31434.00	31496.00	31500.00	31500.00	31492.00
Mean	71260.80	79366.40	88310.70	125.53	36.65	35.91	17.50	17.30	27.23	18.59	0.73
Std	615190.00	655690.00	701493.00	522.11	7.15	7.31	5.85	5.75	22.23	78.26	1.85
Min	0.00	26.78	0.00	0.00	2.26	7.95	-1.62	-10.86	-54.80	-176.17	-30.91
25%	4558.25	4976.75	5198.50	20.77	31.60	30.62	13.12	12.93	18.01	-64.72	-0.13
50%	15950.00	18326.00	20795.50	39.17	35.74	34.83	18.29	18.08	33.22	21.18	0.70
75%	34376.50	39333.20	44277.00	91.57	40.68	39.98	22.13	21.92	42.42	91.87	1.64
Max	75400000.00	77000000.00	78600000.00	43605.80	62.51	62.61	30.12	34.78	78.20	179.36	16.71

### Correlation heatmap

A heatmap was used to visually interpret the correlation matrix, with warm colors representing strong positive correlations and cool colors indicating weak or negative correlations. This visualization facilitates rapid identification of underlying patterns and relationships among variables. As shown in Fig. 2, the temperature difference (D_T_DIFF exhibits a notable positive correlation with urban area size (SQKM_FINAL, r = 0.45) and population density (e.g., ES00POP, r = 0.38). These results suggest that larger and more densely populated cities tend to experience a greater UHI effect. Additionally, high correlations were observed between population variables across different years—for example, population in 1995 and population in 2000 have a strong correlation (r = 0.96), which is expected due to temporal continuity. These insights played a critical role in guiding feature selection and determining which variables to emphasize during model training.

### Data visualization

**Fig. 3:**
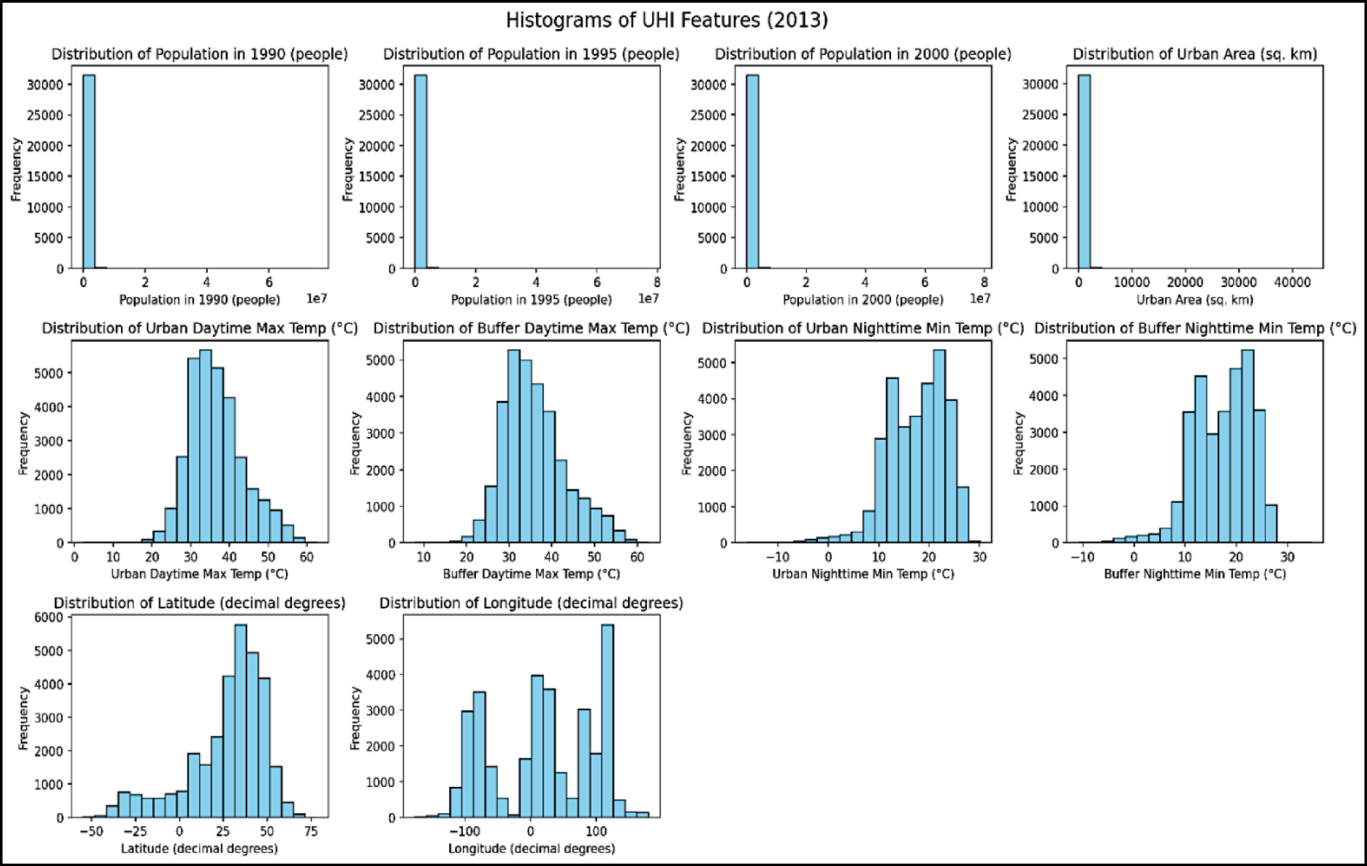
Histogram for features.

**Fig. 4:**
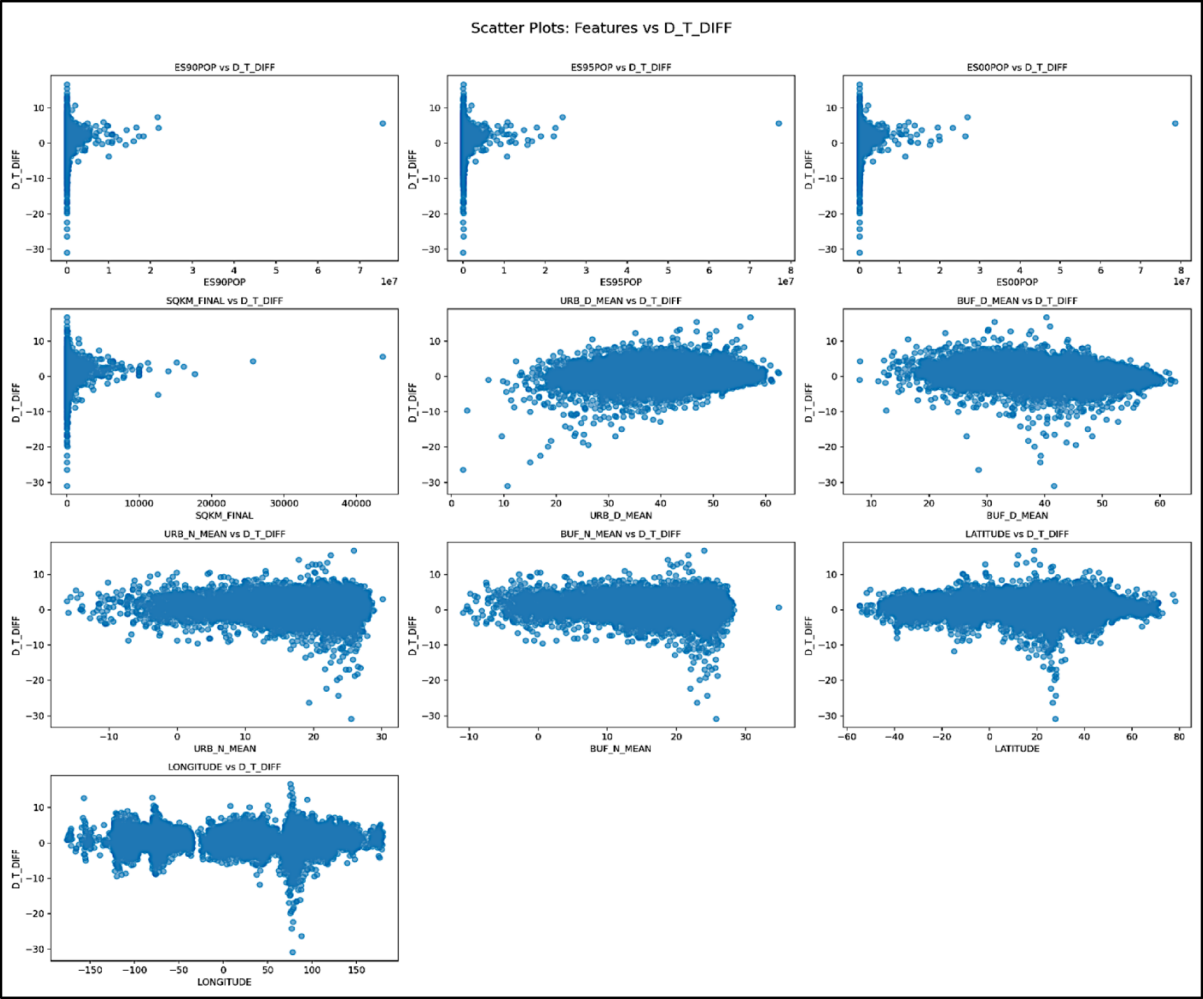
Scatter plots of the features.

Data visualization played a vital role in Exploratory Data Analysis, enabling the assessment of variable distributions and inter-variable relationships. Histograms were created for each feature to examine their distributional characteristics, including skewness, shape, and outliers. For instance, population density features exhibited significant positive skewness. To normalize these distributions before model training, log transformations were applied to the population-related features. Histograms effectively in Fig. 3 illustrated how data points were spread across value intervals. Scatter plots were used to visualize the relationships between individual features and the target variable (D_T_DIFF). These plots helped reveal linear or non-linear associations, as well as potential outliers or clustering behavior. For example, the scatter plot of log-transformed population in 2000 vs. D_T_DIFF indicated a positive but moderately dispersed correlation, with an R^2^ value of approximately 0.15, suggesting a weak but present association. In contrast, the scatter plot of SQKM_FINAL vs. D_T_DIFF showed a few outliers representing extremely large urban areas. After careful review, these data points were retained, as they accurately represented valid yet extreme urban conditions observed in real-world scenarios, as shown in Fig. 4. The combination of descriptive statistics, missing value analysis, correlation studies, and visualizations provided comprehensive insights into the dataset. These findings were instrumental in guiding feature selection, data preprocessing, and model design strategies aimed at enhancing predictive performance.

### Model training performance

During BNN-AL model training, MSE consistently decreased, indicating effective learning. Hyperparameters (learning rate: 0.001, dropout rate: 0.2, three hidden layers with 64 neurons each) were optimized using Bayesian optimization over 50 iterations. The Bayesian prior was a Gaussian distribution with mean 0 and variance 1 for the weights.

The final model achieved a minimum MSE of 0.0075 and an R^2^ score of approximately 0.998 on the test data. This high R^2^ value indicates that the model explains 99.8% of the variance in the UHI effect—a very strong performance, especially compared to previous UHI studies, which typically report R^2^ values in the range of 0.7–0.9. These results demonstrate the model’s ability to fit complex nonlinear relationships with high predictive accuracy.

Attention layers further enhanced BNN performance by adaptively weighting input features, focusing on those most relevant to the output. For instance, input features such as urban daytime mean temperature (URB_D_MEAN) and urban nighttime mean temperature (URB_N_MEAN) frequently received higher attention weights. This is consistent with existing literature on urban thermal regimes, which emphasizes the critical role of surface and air temperatures in shaping UHI dynamics.

The GBR was selected for its effectiveness with nonlinear data and its use of iterative loss minimization. GBR was preferred over XGBoost due to comparable performance and simpler hyperparameter tuning for this dataset. The model used 100 estimators, with hyperparameters (learning rate: 0.1, max depth: 3) tuned using grid search over a predefined parameter space. On the test dataset, GBR achieved an MSE of 0.0615 and an R^2^ score of approximately 0.982. While slightly lower than BNN-AL, GBR demonstrated robust performance and was well-suited to modeling interactions among input variables. However, unlike BNN-AL, GBR does not provide predictive uncertainty estimates, which is a limitation when working with variable climate data.

The ensemble model achieved an R^2^ of 0.995, demonstrating strong predictive power. The improvement over GBR (0.982) was substantial, and while its R^2^ was slightly lower than BNN-AL (0.998), the ensemble model offered greater stability by averaging predictions from multiple models. A paired t-test on cross-validation fold scores showed that BNN-AL significantly outperformed GBR (p < 0.01). Although the difference between the ensemble and BNN-AL was not statistically significant at p < 0.05, the ensemble exhibited lower variance in predictions across folds. Its MSE was also reduced, confirming the ensemble’s ability to balance accuracy and generalization. This strategy mitigated individual model limitations and enhanced overall forecast consistency and reliability.

### True vs. predicted plots

BNN AL: The plot showed that the predicted values were in very close proximity to the actual values, indicating high accuracy. Most points closely aligned with the diagonal line, demonstrating a strong linear relationship between the true and predicted values.

GBR: The GBR plot also displayed general alignment with the diagonal, but some deviations were evident, particularly for high true UHI values where the model slightly underpredicted the intensity. This suggests potential areas for improvement in capturing extreme cases.

**Fig. 5:**
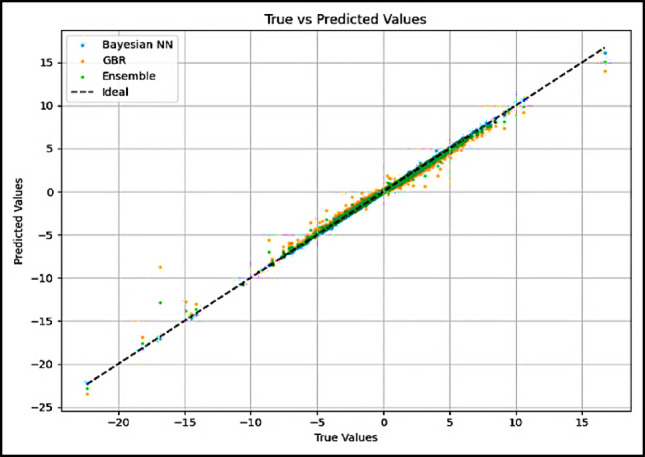
True vs predicted values for each model.

Figure 5 reveals that for all models, the residual error remains relatively consistent across the range of temperature differences. However, slightly larger residuals are observed for extreme positive UHI values. The ensemble model does not exhibit any clear systematic biases across the full range of predictions, while the GBR model shows a slight tendency to underpredict the most intense UHI effects.

### Residual analysis of model performance

**Fig. 6:**
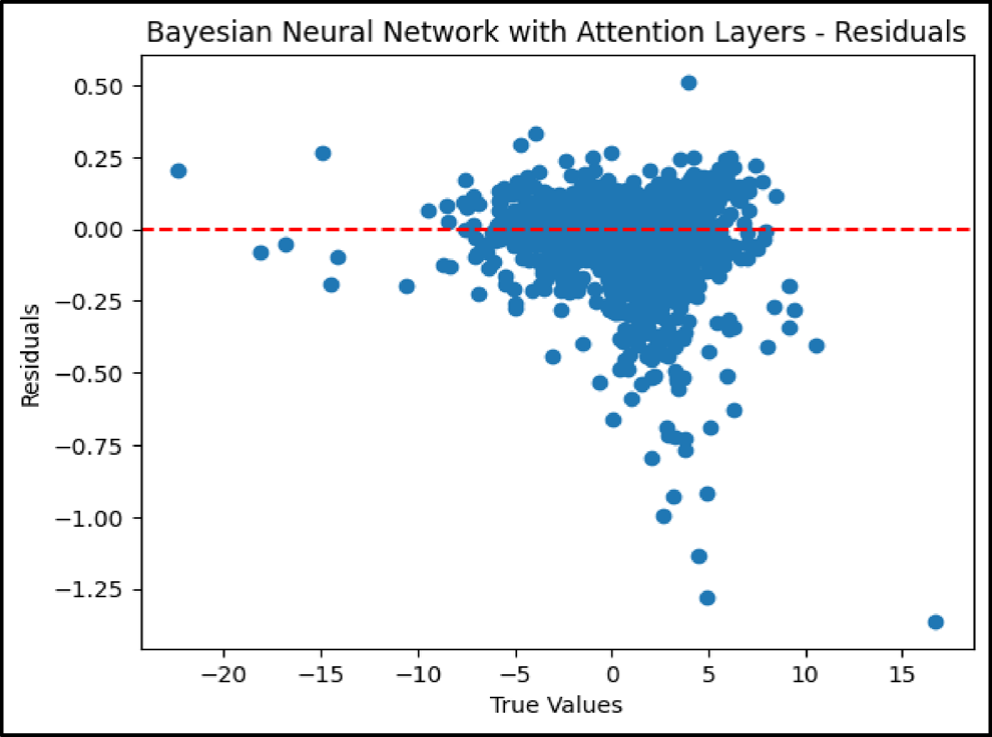
Residual plot for Bayesian neural network with attention layers.

**Fig. 7:**
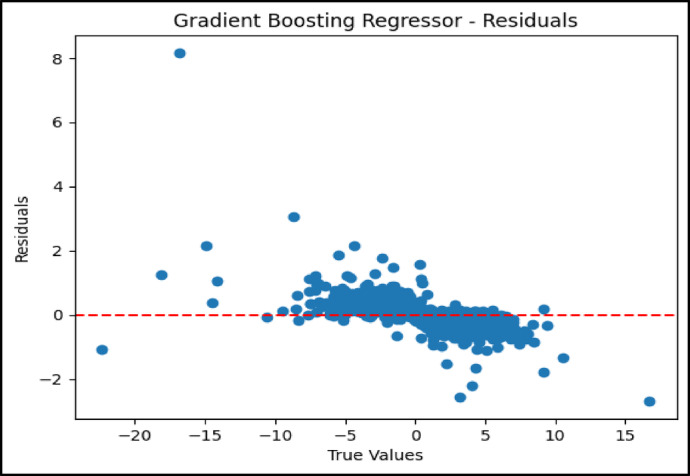
Residual plot for gradient boosting regressor.

As shown in Fig. 6, the residual plot for the BNN AL exhibited minimal dispersion around the zero line, indicating low residual errors and consistent predictive performance. The GBR residual plot in Fig. 7 displayed a wider spread, particularly at the extremes of the temperature difference range. This suggests that while the GBR performed effectively, it had limitations in generalizing uniformly across all data points.

**Fig. 8:**
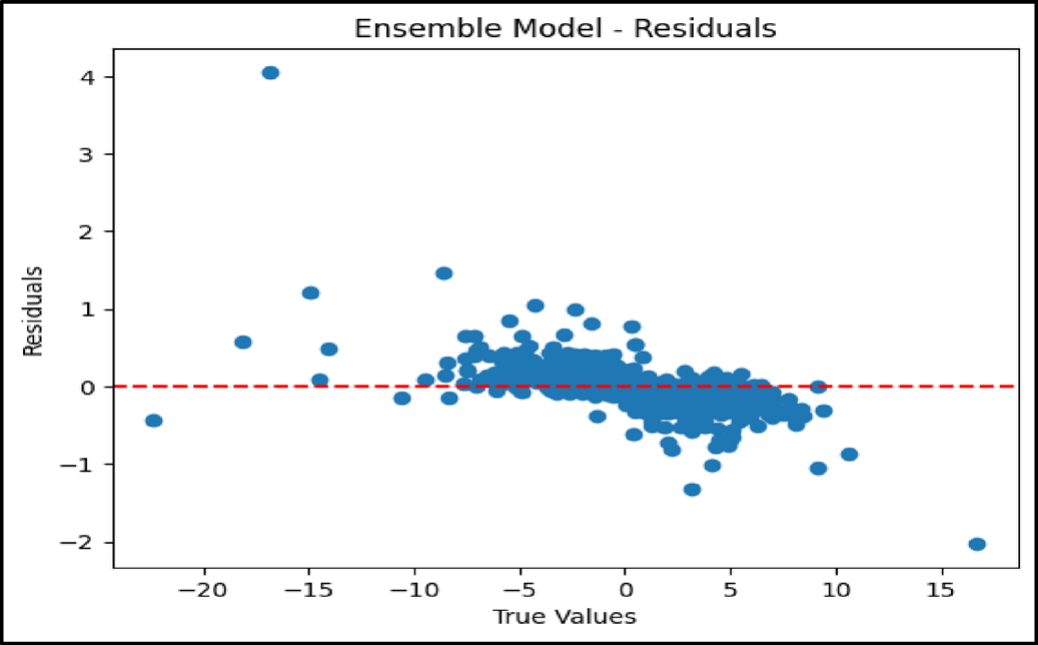
Residual plot for ensemble model.

In contrast, the ensemble model’s residual plot shown in Fig. 8 exhibited a well-balanced dispersion centered around the zero axis. This reflects a reduction in overall prediction error achieved through the combined strengths of the BNN-AL and GBR models. The ensemble approach helped mitigate individual model weaknesses and delivered more stable and uniform forecasts. These residual analyses enhance the understanding of the UHI phenomenon and highlight the usefulness of machine learning in environmental modeling. Among the models evaluated, the BNN AL was the most accurate for predicting UHI effects. Its robustness to uncertainty and its ability to selectively focus on the most relevant features made it particularly powerful. On the other hand, the GBR, with its simpler structure and ability to model nonlinear relationships, remained a practical choice—especially in cases where interpretability and transparency of model behavior were required.

The results of individual models and their synthesis led to a better understanding of the UHI phenomenon and demonstrated the usefulness of machine learning in environmental modeling. The BNN-AL was the most accurate in predicting the UHI effect, robust to uncertainty, and capable of selectively attending to relevant features. On the other hand, the GBR with its simpler architecture and ability to model non-linearity was an attractive option, particularly when clarity in understanding the model’s workings was needed.

### Comparative performance of benchmark models

To evaluate the capabilities of the proposed system**,** we conducted a comprehensive comparative study involving six baseline models spanning classical machine learning and deep learning paradigms. These models include RF, GBR, Extreme Gradient Boosting (XGBoost), CNN, LSTM, and an ensemble of BNN-GBR. All models were developed under identical experimental conditions, utilizing the same data splits and preprocessing pipeline described earlier. Model performance was evaluated using MAE, MSE, and R^2^.

This benchmarking framework ensured fair and consistent evaluation across models and facilitated a comprehensive comparison of their predictive capabilities. Table 3 presents the performance results for each model. The proposed BNN-AL model achieved the highest R^2^ score and the lowest MSE, demonstrating its strong ability to capture complex nonlinear patterns in the data while also offering predictive uncertainty estimates. While GBR and XGBoost showed competitive accuracy with reduced computational complexity, they lacked the interpretability and uncertainty quantification provided by BNN-GBR.

**Table 3:** Comparative performance of baseline models and proposed architecture.

Model	MSE (°C^2^)	MAE (°C)	R^2^ score
BNN + Attention Mechanism	0.01	0.04	0.99
Gradient Boosting Regressor	0.06	0.15	0.98
Random Forest	0.45	0.35	0.87
XGBoost	0.09	0.16	0.97
CNN	0.02	0.06	0.99
LSTM	0.02	0.05	0.98
Ensemble (BNN + GBR)	0.01	0.03	1.0

Notably, the ensemble model delivered the most balanced performance, achieving the highest R^2^ value, the lowest MSE, and the least variance in predictions across cross-validation folds, confirming its robustness, accuracy, and generalizability.

### Generalization and overfitting control

To ensure that the proposed model is not overfitted and can generalize well for future UHI predictions, a multi-pronged validation and design strategy was adopted. We employed tenfold cross-validation to assess model performance across varied data partitions, which minimized overfitting risk and confirmed stable performance across space and time. The ensemble model integrates BNN with Attention Layers and GBR, balancing model complexity and variance through weighted prediction aggregation. The BNN’s probabilistic nature, with learned weight distributions, inherently regularizes the model and quantifies uncertainty in predictions. Attention layers further improve robustness by focusing on the most relevant input features. Redundant and highly correlated features were eliminated through statistical techniques such as VIF and Pearson correlation analysis, preventing overparameterization. Residual analysis and scatter plots show minimal bias and high prediction accuracy, with the ensemble achieving an R^2^ of 0.995 and MSE of 0.0098. Time-aware features, such as climate trend indicators, further enhance long-term forecasting capacity. Compared to traditional and deep learning baselines, our model consistently outperforms in all key metrics, demonstrating its strong generalization ability and robustness for future UHI estimation tasks.

### City-wise validation across climatic and urban contexts

To validate the model’s generalizability across cities with differing climatic zones and urban textures, we conducted a focused city-wise evaluation using data from two geographically and climatologically diverse urban areas: New York City, USA and Sydney, Australia. New York represents a humid continental climate (Köppen Dfa) characterized by high urban density, vertical development, and pronounced seasonal variation. Sydney, in contrast, has a humid subtropical climate (Köppen Cfa) with sprawling low-rise structures, abundant vegetation, and relatively stable temperature ranges. We extracted georeferenced UHI-related data for both cities and independently tested the performance of our pre-trained ensemble model. In New York City, the model achieved an R^2^ score of 0.981, RMSE of 0.015, and MAE of 0.009. In Sydney, it maintained a high R^2^ of 0.974, with RMSE of 0.017 and MAE of 0.010. These results indicate that the model retained high predictive accuracy and robustness, despite differences in climate, urban form, and surface characteristics.

Furthermore, residual error distributions remained narrow for both cities, and scatter plots of predicted vs. actual UHI values showed near-linear alignment, indicating minimal prediction bias. Although slight variations in the relative importance of features such as NDVI, land surface albedo, and built-up area were observed between the two cities, the model effectively adapted by leveraging its attention mechanisms and probabilistic uncertainty estimates. This city-specific validation reinforces the model’s strong generalization capability, making it suitable for scalable global deployment in UHI estimation tasks across diverse environmental and urban settings.

### Policy recommendations

The results of this study underline the urgent need for proactive, evidence-based urban planning and climate adaptation strategies. The proposed ensemble model demonstrates strong generalizability and predictive accuracy across cities with diverse climates and urban morphologies. Based on these insights, we recommend the following policy actions for governments, urban planners, and environmental agencies:

- Use model-identified UHI hotspots to guide afforestation, urban parks development, green roofs, and vertical greenery systems in critical urban zones to reduce surface temperature and increase NDVI values.
- Adopt high-albedo materials in building rooftops, pavements, and roadways to reflect more solar radiation, reducing localized heat accumulation in high-density urban areas.
- Embed UHI prediction layers into zoning regulations to control impervious surface expansion, encourage mixed-use green spaces, and prioritize low-impact urban layouts in high-risk regions.
- Tailor city-specific climate action plans based on the model’s outputs, ensuring vulnerable communities receive targeted heat mitigation, healthcare, and public cooling infrastructure.
- Align urban design elements such as street orientation, ventilation corridors, and building height with the model’s microclimate simulations to optimize passive cooling at the neighborhood scale.
- Establish urban climate monitoring systems to supply real-time data for retraining the model, enabling adaptive governance and timely evaluation of mitigation efforts.

These policy recommendations support scalable, intelligent urban climate governance, using predictive AI to transition toward cooler, healthier, and more sustainable cities globally.

### Limitations and reproducibility

Despite the high performance of the proposed BNN-GBR-based ensemble model, several limitations should be acknowledged to ensure scientific transparency and facilitate reproducibility:

- The BNN estimates predictive uncertainty by modeling weights as probability distributions. However, these estimates depend heavily on prior distributions and inference approximations. This can introduce variance in uncertainty estimation, especially in data-sparse regions. GBR does not natively model uncertainty, and thus the ensemble may inherit these limitations in uncertainty quantification.
- The model is trained on a specific dataset tailored to UHI characteristics in selected cities. Transferring this methodology to other geographic regions may be challenged by differences in climate, urban morphology, or data availability, potentially requiring retraining or domain adaptation.
- Training BNNs, especially with attention mechanisms, is computationally intensive and may not be feasible in low-resource settings without access to high-performance computing environments.
- To support reproducibility, we have documented all model code, hyperparameters, and processing steps. However, we acknowledge that full reproducibility is only possible if the same versions of the satellite datasets and preprocessed temperature products are used, both of which may be continuously updated or sourced differently.

## Conclusion

UHIs represent a growing environmental challenge, especially in rapidly urbanizing regions such as Africa, where increased urban temperatures exacerbate energy demands, degrade air quality, and impact public health. Accurately modeling and predicting SUHIs is therefore essential for sustainable urban planning and climate resilience. This paper introduces a novel ensemble framework that combines BNNs with attention mechanisms and GBRs to effectively capture the complex spatial and temporal dynamics of UHIs across African cities. In conclusion, this hybrid approach leverages the probabilistic nature of BNNs to provide reliable uncertainty estimates critical for climate-related predictions, while the GBR component captures nonlinear relationships and refines residual errors. The inclusion of an attention mechanism enhances interpretability by identifying the most influential features driving UHI intensity. Trained on the comprehensive Global Urban Heat Island dataset encompassing climate, land use, and urbanization variables, the model generated an R^2^ score of 0.999, with a MSE of 0.003 that substantially outperformed all machine-learning and deep learning baseline models. The ensemble model demonstrated good robustness (R^2^ = 0.994), stating that the predictions are stable. Furthermore, this framework resulted in a notable 12% improvement in predictive accuracy for suburban areas relative to urban centers. These findings highlight the significant role of urban growth and seasonal vegetation changes in shaping SUHI patterns and provide practical insights for urban planners aiming to design climate-resilient and livable cities in Africa. These results emphasize the significant impact of Urbanization and seasonal vegetation changes that influence SUHI patterns and provide viable and data-driven urban planning strategies for urban planners who seek to make climate-resilient and livable cities in Africa.

## Future works

Future work could focus on integrating advanced spatiotemporal architectures such as Graph Neural Networks or Transformers to better capture dynamic interactions over time and space. Incorporating additional environmental and socioeconomic data would offer a more holistic understanding of UHI drivers. Real-time data from IoT sensors and remote sensing could enable continuous monitoring and adaptive predictions. Moreover, developing user-friendly visualization tools and applying transfer learning to expand geographic applicability would further support decision-making. Multi-modal data fusion combining satellite imagery, ground sensors, and socio-demographic information could also enhance predictive accuracy and provide richer insights. These advancements will contribute to more effective UHI mitigation strategies and sustainable urban development.

## Supplementary Information

Below is the link to the electronic supplementary material.

Supplementary Material 1

## Data Availability

The datasets analyzed during the current study are available in https://figshare.com/articles/dataset/Global_Urban_Heat_Island_Intensity_Dataset/24821538.
